# Supplementary material for: Elevated PDK1 Expression Drives PI3K/AKT/MTOR Signaling Promotes Radiation-Resistant and Dedifferentiated Phenotype of Hepatocellular Carcinoma
Source: Cells. 2020 Mar 18;9(3):746. doi: 10.3390/cells9030746 (PMC7140693; doi:10.3390/cells9030746)
Supplement: Supplementary file 1 [file cells-09-00746-s001.pdf]

SUPPLEMENTARY INFORMATION

Elevated PDK1 expression drives PI3K/AKT/MTOR signaling promotes radiation-resistant and dedifferentiated phenotype of hepatocellular carcinoma

Oluwaseun Adebayo Bamodu [1,2#], Hang-Lung Chang [3,4#], Jiann-Ruey Ong[5,6], Wei-Hwa Lee[7], Chi-Tai Yeh[1,2,8], Jo-Ting Tsai[9,10,11*]

[1] Department of Hematology and Oncology, Cancer Center, Taipei Medical University - Shuang Ho Hospital, New Taipei City 235, Taiwan; [2] Department of Medical Research and Education, Taipei Medical University - Shuang Ho Hospital, New Taipei City 235, Taiwan; [3] Department of General Surgery, En Chu Kong Hospital, New Taipei City 237, Taiwan; [4] Department of Health Care Management, Yuanpei University of Medical Technology, Hsinchu 300, Taiwan; [5] Department of Emergency Medicine, School of Medicine, Taipei Medical University, Taipei City 110, Taiwan; [6] Department of Emergency Medicine, Taipei Medical University – Shuang Ho Hospital, New Taipei City 235, Taiwan; [7] Department of Pathology, Taipei Medical University-Shuang Ho Hospital, New Taipei City 235, Taiwan. [8] Department of Medical Laboratory Science and Biotechnology, Yuanpei University of Medical Technology, Hsinchu City 300, Taiwan. [9]Department of Radiology, School of Medicine, College of Medicine, Taipei Medical University, Taipei City 110, Taiwan; [10] Department of Radiology, Taipei Medical University - Shuang Ho Hospital, New Taipei City 235, Taiwan. [11] Graduate Institute of Clinical Medicine, College of Medicine, Taipei Medical University, Taipei City 110, Taiwan.

# Contributed equally to this work

*Corresponding author(s):

Jo-Ting Tsai, MD., PhD. Department of Radiation Oncology, Cancer Center, Taipei Medical University - Shuang Ho Hospital, New Taipei City 23561, Taiwan; Tel: +886-2-2490088 ext. 8885, Fax: +886-2-2248-0900, E-mail: 10576@s.tmu.edu.tw

Supplementary Table S1. Antibody list.

No.	Target	Dilution	Catalog	kDa
**1**	p-PI3K	1:1000	Phospho-PI3 Kinase p85 (Tyr458)/p55 (Tyr199) Antibody #4228S	60 and 85
**2**	PI3K	1:1000	PI3 Kinase p110α (C73F8) Rabbit mAb #4249S	110
**3**	p-PDK1	1:1000	Phospho-PDK1 (Ser241) Antibody #3061S	58 - 68
**4**	PDK1	1:1000	PDK1 (D37A7) Rabbit mAb #3062S	58 - 68
**5**	p-AKT	1:1000	Phospho-Akt (Ser473) (D9E) XP® Rabbit mAb #9271L	60
**6**	AKT	1:1000	Akt Rabbit Polyclonal antibody #4691L	60
**7**	p-mTOR	1:1000	Phospho-mTOR (Ser2481) Antibody#2971L	289
**8**	mTOR	1:1000	mTOR (7C10) Rabbit mAb #2983	289
**9**	β-actin	1:10000	β-Actin (8H10D10) Mouse mAb sc-69879	42
**10**	E-Cadherin	1:1000	E-Cadherin (24E10) Rabbit mAb #3195	125-135
**11**	N-cadherin	1:1000	N-Cadherin Rabbit polyclonal Antibody #13116S	140
**12**	Vimentin	1:1000	Anti-Vimentin antibody (ab137321) #5741S	57
**13**	Snail	1:1000	Snail (C15D3) Rabbit mAb #3879S	29
**14**	Bax	1:1000	Bax (D2E11) Rabbit mAb #5023S	20
**15**	Bcl-2	1:1000	Bcl-2 (50E3) Rabbit mAb#15071S	26
**16**	SOX2	1:1000	Anti-SOX2 antibody (ab97959) #3579S	37
**17**	OCT4	1:1000	Oct-4 Antibody #2750 #2840S	45
**18**	ALDH1	1:1000	ALDH1A1 (D4R9V) Rabbit mAb #12035	55

A

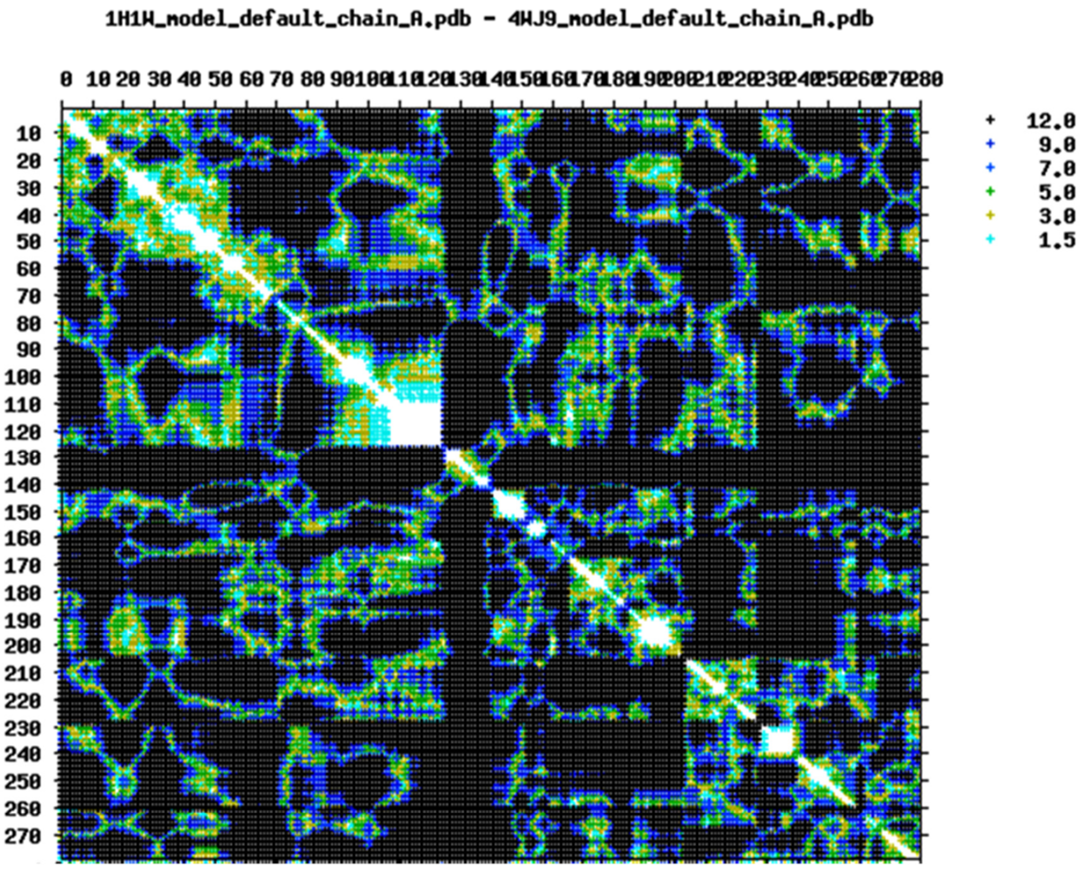

Local RMSD

	Alpha Carbons	Back Bone	Heavy	All
RMSD	23.54	23.57	23.52	23.52
Atoms	279	1116	1575	1575

Structure	Residues
1H1W chain 'A'	71-86, 87-139, 145-199, 200-214, 215-226, 227-232, 237-237, 238-240, 242-244, 245-263, 264-279, 280-282, 283-296, 297-306, 307-318, 319-338, 339-340, 341-345, 346-359
4WJ9 chain 'A'	11-26, 29-81, 82-136, 142-156, 160-171, 192-197, 198-198, 208-210, 211-213, 219-237, 243-258, 266-268, 275-288, 292-301, 313-324, 329-348, 366-367, 371-375, 384-397

Global RMSD

	Alpha Carbons	Back Bone	Heavy	All
RMSD	23.54	23.57	23.52	23.52
Atoms	279	1116	1575	1575

Structure	Residues
1H1W chain 'A'	71-86, 87-139, 145-199, 200-214, 215-226, 227-232, 237-237, 238-240, 242-244, 245-263, 264-279, 280-282, 283-296, 297-306, 307-318, 319-338, 339-340, 341-345, 346-359
4WJ9 chain 'A'	11-26, 29-81, 82-136, 142-156, 160-171, 192-197, 198-198, 208-210, 211-213, 219-237, 243-258, 266-268, 275-288, 292-301, 313-324, 329-348, 366-367, 371-375, 384-397

*PDK1 : 1H1W (PDB ID); ALDH1:4WJ9 (PDB ID)

B

Sequence alignment

```
1H1W_model_de    1 PPQPRKKRPEDFKFGKILGEGSFSTVVLARELATSREYAIKILEKRHIIK     50

4WJ9_model_de    0 --------------------------------------------------      0

1H1W_model_de   51 ENKVPYVTRERDVMSRLDHPFFVKLYFTFQDDEKLYFGLSYAKNGELLKY    100

4WJ9_model_de    0 --------------------------------------------------      0

1H1W_model_de  101 IRKIGSFDETCTRFYTAEIVSALEYLHGKGIIHRDLKPE--NILLNEDMH    148
                                               ..::..|||.:  .|.:|.:.|
4WJ9_model_de    1 ----------------------------LPVLLTDLKIQYTKIFINNEWH     22

1H1W_model_de  149 IQITDFGTAK---VLSPARANFVGTAQYVSPELLTEKSACK-----SSDL    190
                       |..:.|   |.:||                ||:..|:     ..|
4WJ9_model_de   23 ----DSVSGKKFPVFNPA----------------TEEELCQVEEGDKED-     51

1H1W_model_de  191 WALGCIIYQLVAGLPPFRAGNEYLIFQKIIKLEYDFPE--KFFPKARDLV    238
                        :...:.|....|:.|:.:...        |..|  :...|..||:
4WJ9_model_de   52 -----VDKAVKAARQAFQIGSPWRTM--------DASERGRLLYKLADLI     88

1H1W_model_de  239 EKLLVLDATKRLGCEEMEGYGPLKAHPFFESVT-----------WENLHQ    277
                   |:..:|.||    .|.|.| |.|.::.:...:.           |.:
4WJ9_model_de   89 ERDRLLLAT----MESMNG-GKLYSNAYLSDLAGCIKTLRYCAGWAD---    130

1H1W_model_de  278 QTPPKLT-------------------------------------------    284
                       |:.
4WJ9_model_de  131 ----KIQGRTIPIDGNFFTYTRHEPIGVCGQIIPWNFPLVMLIWKIGPAL    176

1H1W_model_de  284 --------------------------------------------------    284

4WJ9_model_de  177 SCGNTVVVKPAEQTPLTALHVASLIKEAGFPPGVVNIVPGYGPTAGAAIS    226

1H1W_model_de  284 --------------------------------------------------    284

4WJ9_model_de  227 SHMDIDKVAFTGSTEVGKLIKEAAGKSNLKRVTLELGGKSPCIVLADADL    276

1H1W_model_de  284 --------------------------------------------------    284

4WJ9_model_de  277 DNAVEFAHHGVFYHQGQCCIAASRIFVEESIYDEFVRRSVERAKKYILGN    326

1H1W_model_de  284 --------------------------------------------------    284

4WJ9_model_de  327 PLTPGVTQGPQIDKEQYDKILDLIESGKKEGAKLECGGGPWGNKGYFVQP    376

1H1W_model_de  284 --------------------------------------------------    284

4WJ9_model_de  377 TVFSNVTDEMRIAKEEIFGPVQQIMKFKSLDDVIKRANNTFYGLSAGVFT    426

1H1W_model_de  284 --------------------------------------------------    284

4WJ9_model_de  427 KDIDKAITISSALQAGTVWVNCYGVVSAQCPFGGFKMSGNGRELGEYGFH    476

1H1W_model_de  284 -----------------    284

4WJ9_model_de  477 EYTEVKTVTVKISQKNS    493
```

Supplementary Figure S1. PDK1 directly binds to and activates ALDH1. (A) ALDH1-PDK1 protein interaction matrix (upper panel) with local and global RMSD data indicated (lower panel). (B) ALDH1-PDK1 protein sequence alignment confirming complementarity.

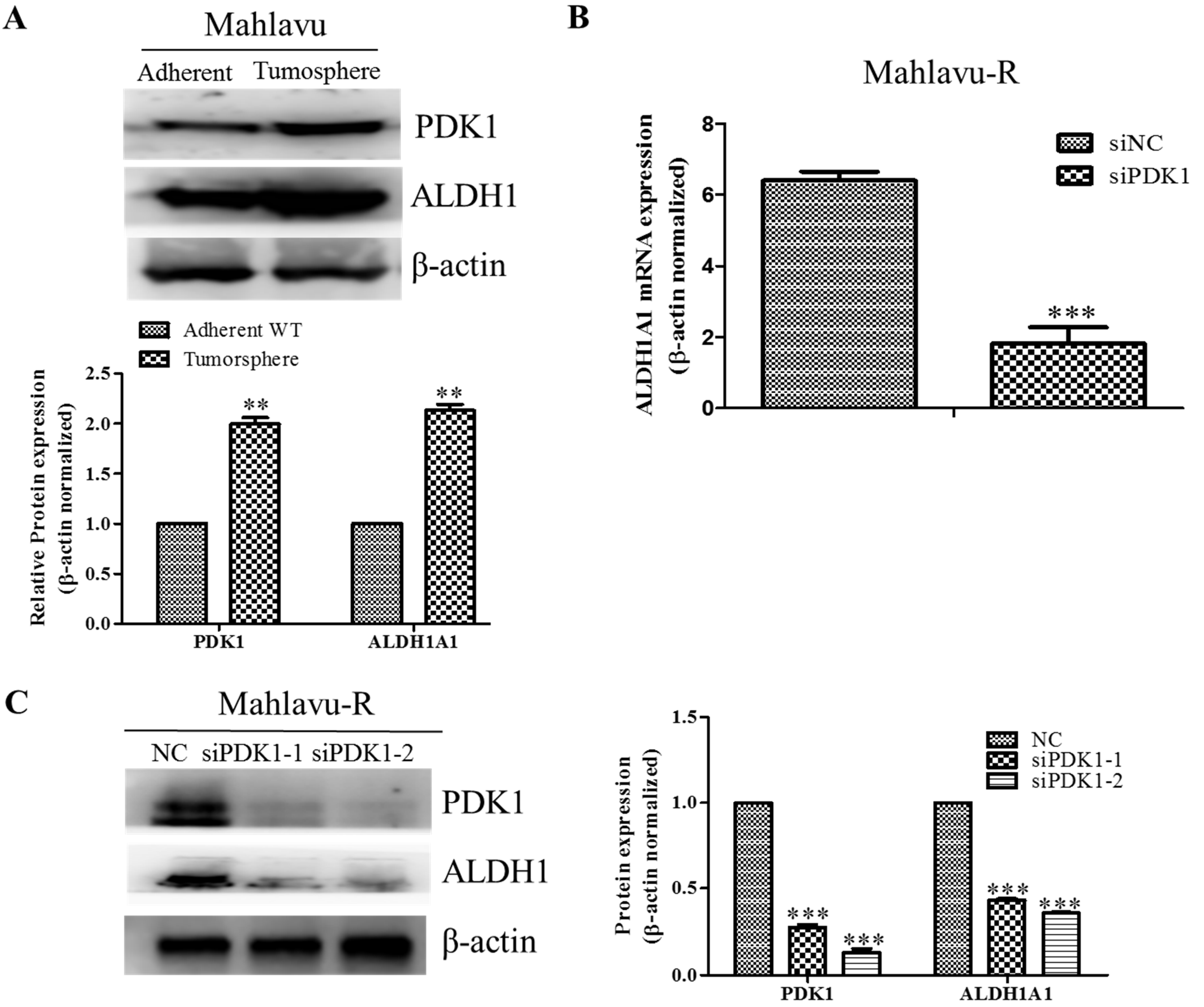

Supplementary Figure S2. PDK1 interacts with ALDH and directly modulate the expression and/or activity of ALDH in HCC cells. (A) Representative western blot image and histograms of the differential expression of PDK1 and ALDH1 in adherent wild-type Mahlavu cells or their tumorsphere counterparts. (B) Graph showing the effect of siPDK1 on the expression level of ALDH1 mRNA in Mahlavu-R cells. (C) Representative western blot image and histograms showing the effect of siPDK1-1 and siPDK1-2 on the expression levels of PDK1 or ALDH1 protein in Mahlavu-R cells. *$p<0.05$, **$p<0.01$, ***$p<0.001$. Mahlavu-R, radioresistant mahlavu cells; WT, wild-type; NC, negative control.

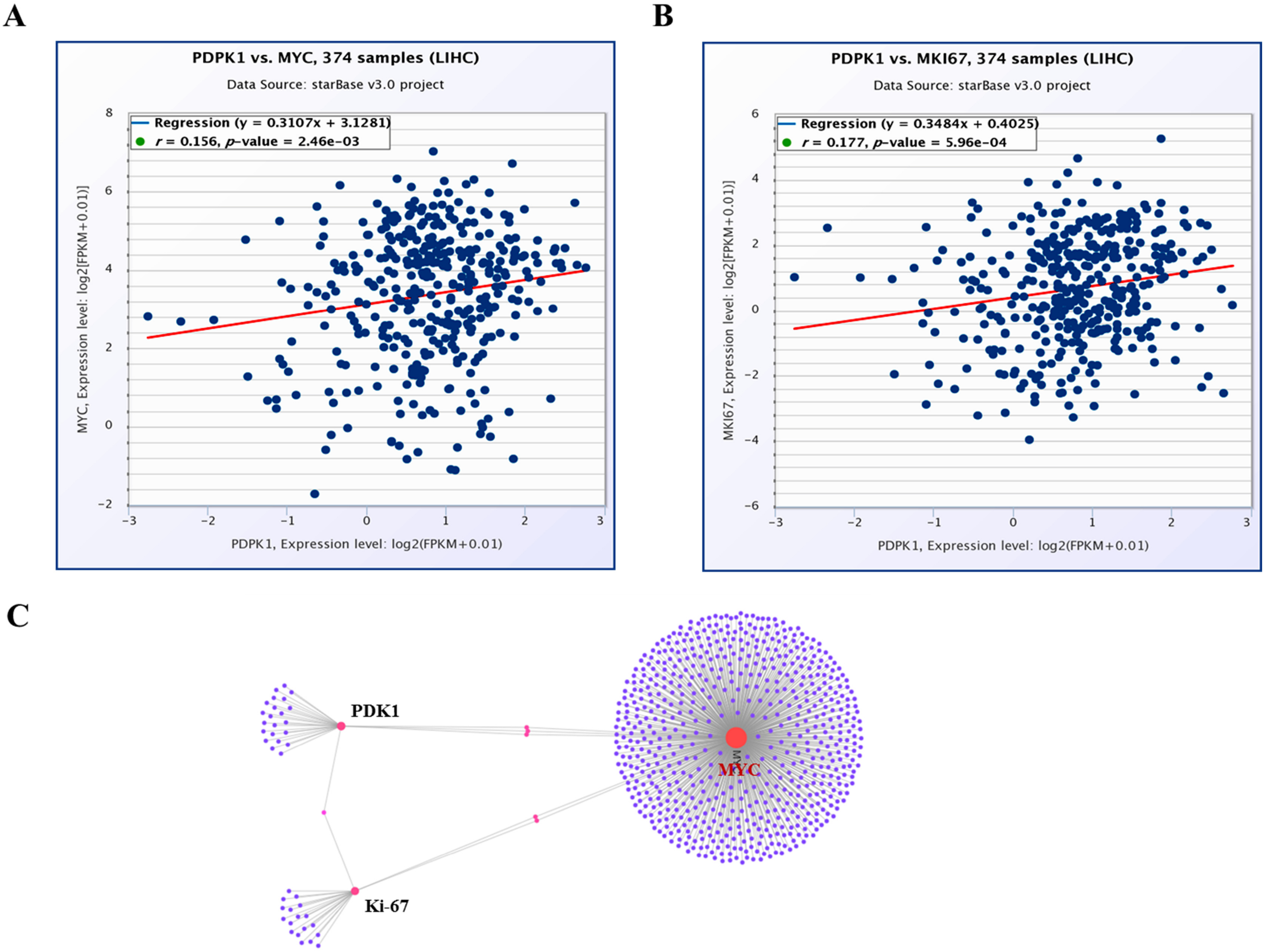

Supplementary Figure S3. PDK1 is associated with the modulation of cellular pluripotency and proliferation. Graphical representation of the correlation between PDK1 and (A) c-MYC/MYC, or (B) Ki-67/MKI67 in the TCGA-liver hepatocellular carcinoma (LIHC) cohort, n = 374. (C) 2D visualization of the protein-protein interaction network between PDK1, MYC, and Ki-67.

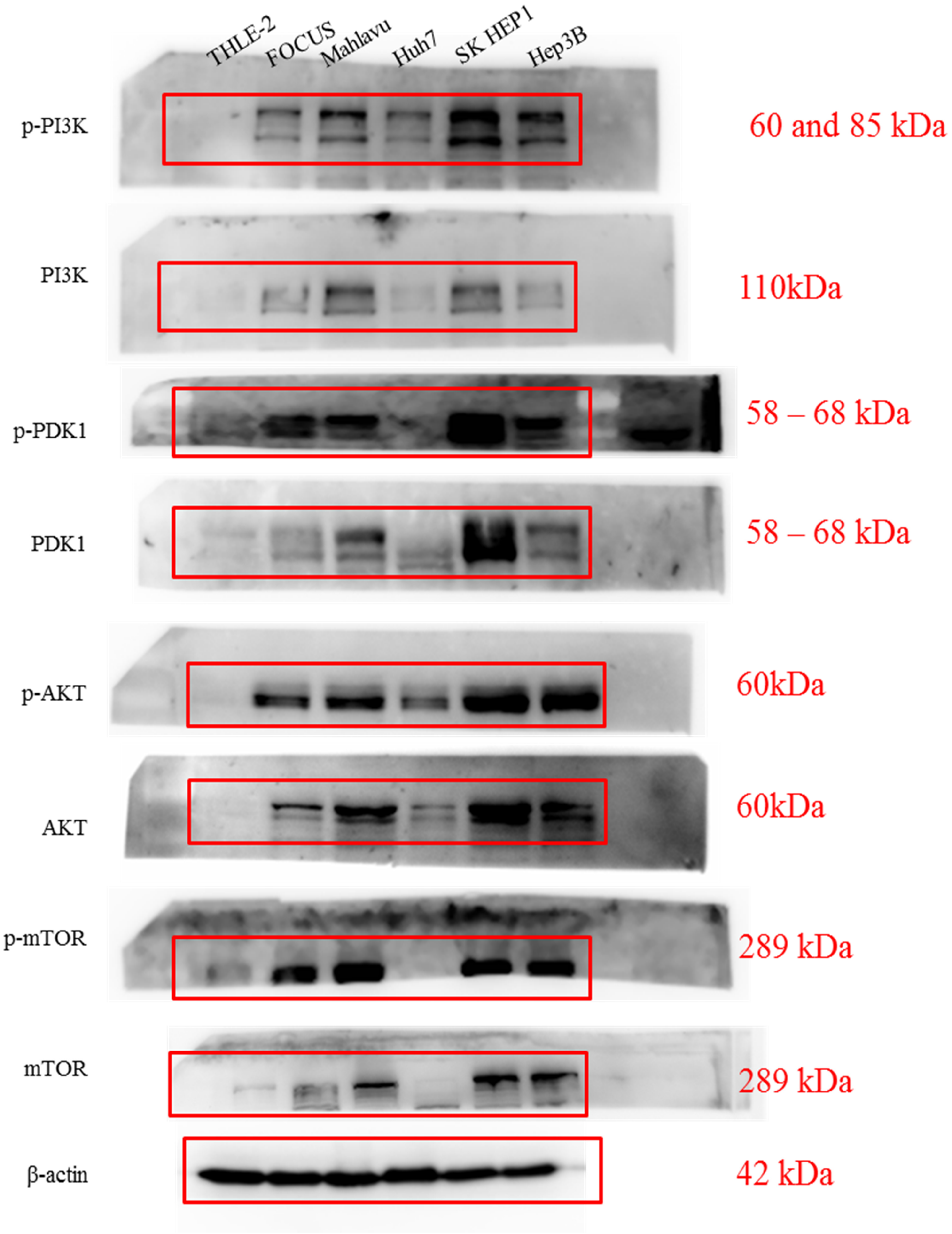

Supplementary Figure S4. Full-size blots of Figure 1C

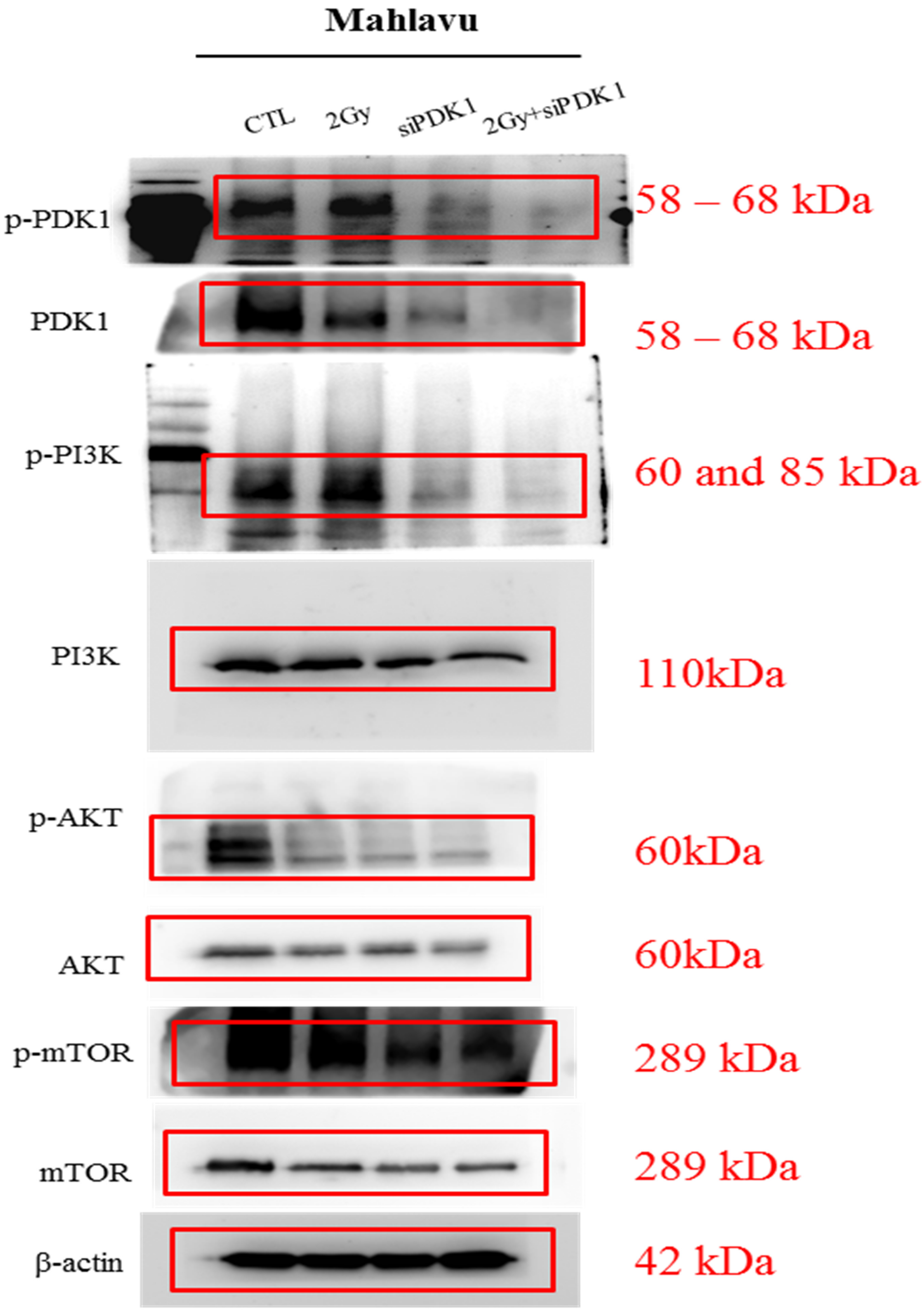

Supplementary Figure S5. Full-size blots of Figure 2D

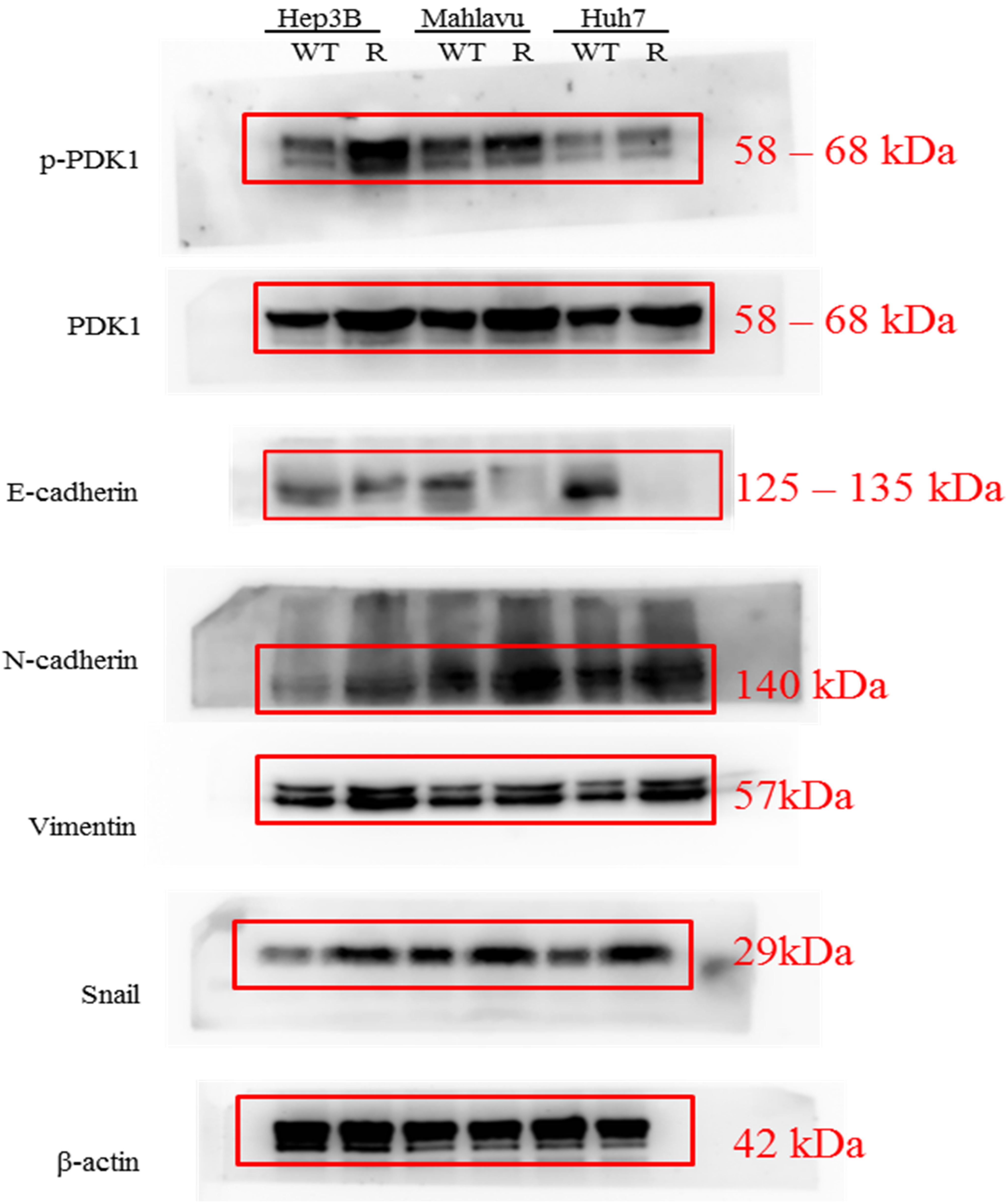

Supplementary Figure S6. Full-size blots of Figure 4D

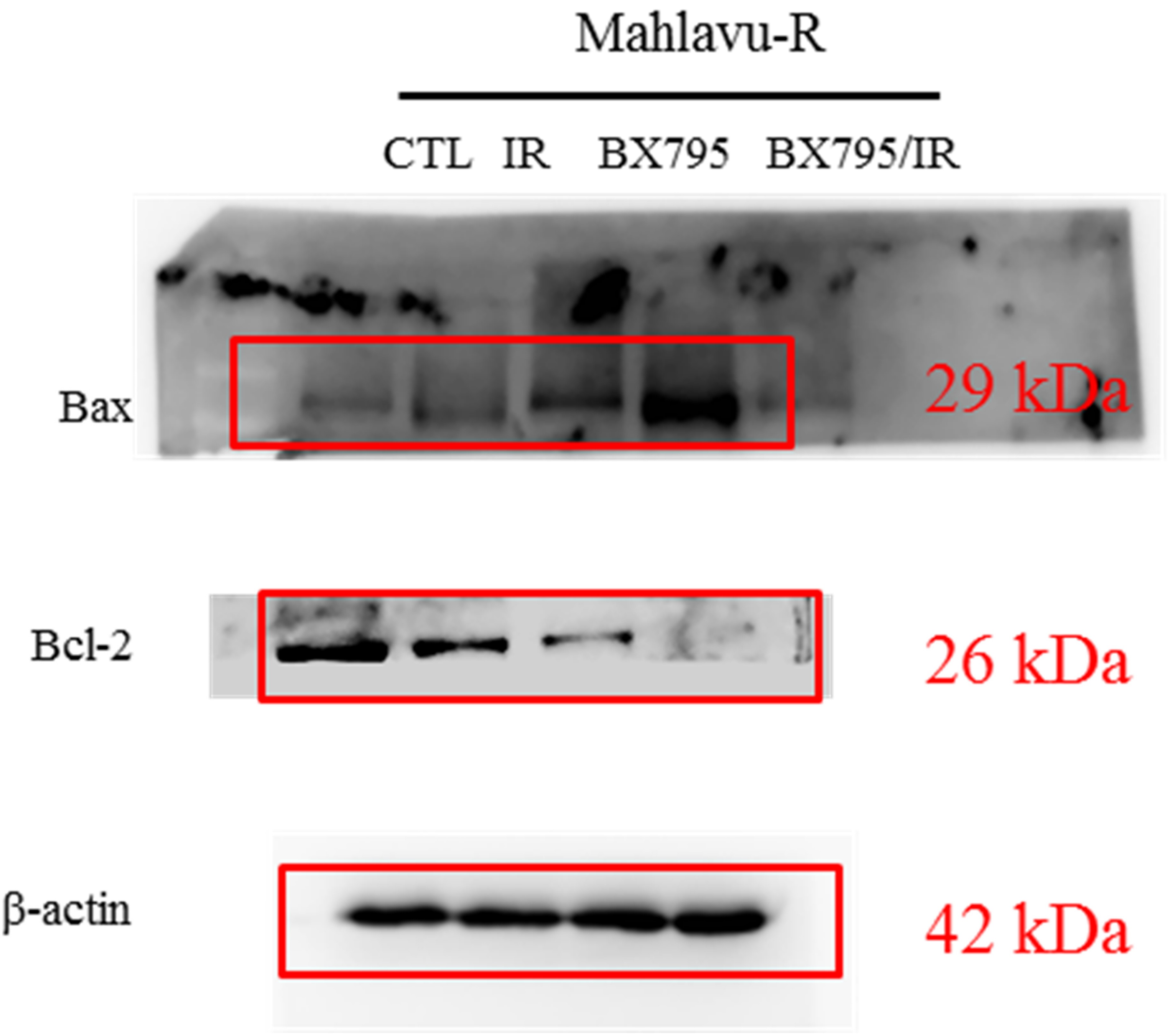

Supplementary Figure S7. Full-size blots of Figure 5C

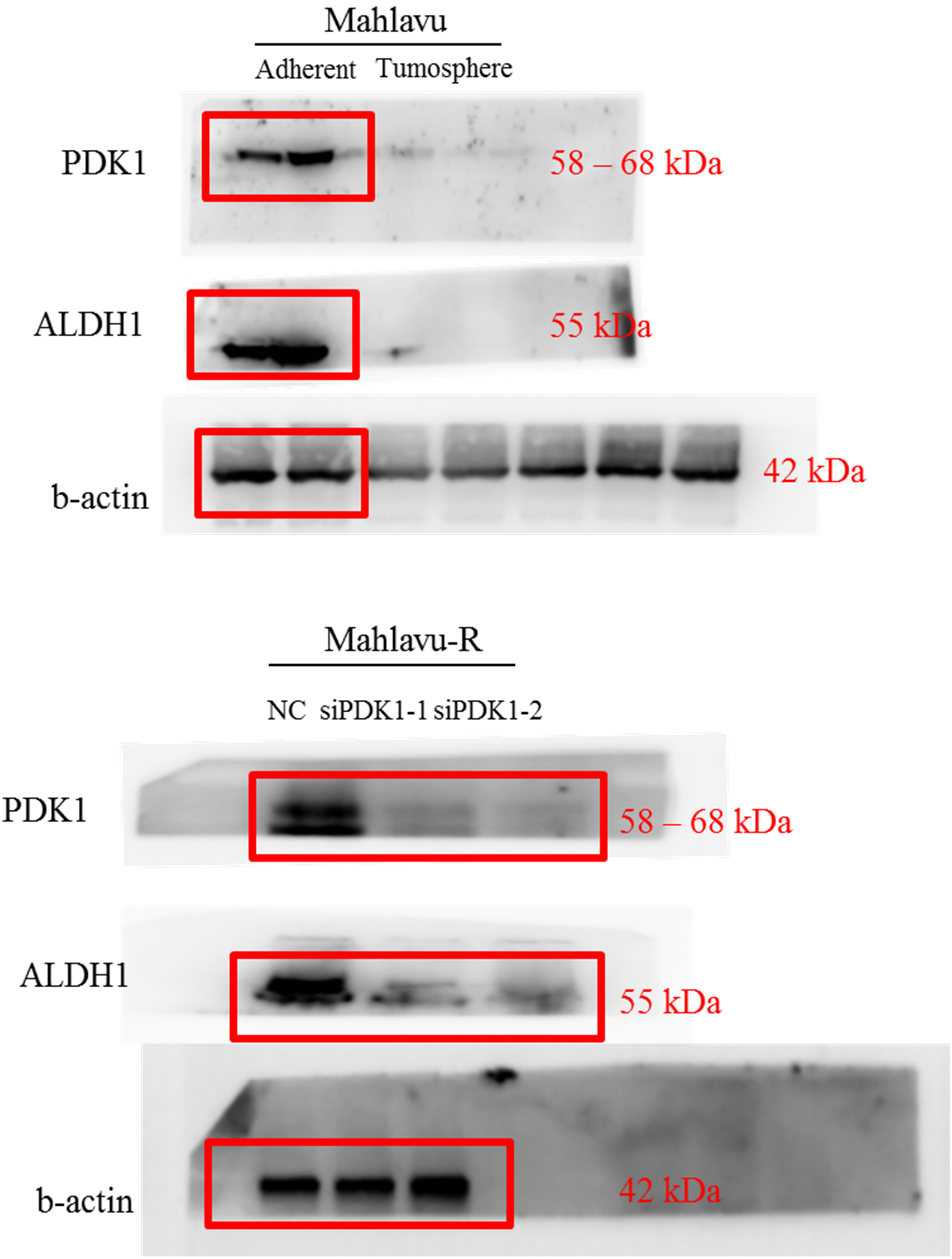

Supplementary Figure S8. Full-size blots of Figure S3 A & B
